# Bulky palpebral Merkel cell carcinoma of the eyelid

**DOI:** 10.3332/ecancer.2025.1946

**Published:** 2025-07-17

**Authors:** Dario Alvaro Rueda, Cecilia Schweitzer, Lorena Di Nisio, María Laura Piccoletti, Nicolas Torressi, Víctor Acevedo, Silvia Ferrandini

**Affiliations:** 1 Clinical Oncology Division, Hospital de Clínicas José de San Martin (UBA), Buenos Aires 1120, Argentina; 2 Oculoplastic Surgery Department, Hospital de Clínicas José de San Martin (UBA), Buenos Aires 1120, Argentina; 3 Otorhinolaryngology Department, Hospital de Clínicas José de San Martin (UBA), Buenos Aires 1120, Argentina; a https://orcid.org/0009-0005-5657-8463

**Keywords:** Carcinoma, Merkel cell/therapy, skin neoplasms/etiology, male, neoplasm staging

## Abstract

Merkel cell carcinoma (MCC) is a rare and aggressive tumour of the skin, characterised by a high rate of local recurrence and lymph node involvement. We present the case of a 58-year-old woman who developed a 5-cm tumour on the right lower eyelid, leading to ocular occlusion. Magnetic resonance imaging revealed an exophytic lesion in the right orbit, and a biopsy confirmed the diagnosis of MCC. After complete surgical resection and cervical emptying, the patient was treated with adjuvant radiotherapy. The final diagnosis was MCC, stage pT3 pN1 M0. The periocular location and tumour size were determinants in the treatment decision.

## Introduction

Merkel cell carcinoma (MCC) is a rare and aggressive tumour of the skin, characterised by a high rate of local recurrence and nodal involvement. Risk factors include advanced age, male gender, white race and immunocompromised status (such as HIV infection, organ transplantation or B-cell neoplasms) [1–3].

MCC was first described in 1972 by Cyril Toker referring to it as ‘trabecular carcinoma of the skin’ [4].

The most frequent location is in the head and neck area (43%) [1–3]. Approximately 65% of cases are diagnosed in localised phase, 26% present locoregional involvement and 8% are metastatic [2].

Locoregional recurrence rates range from 30% to 60%, and up to one-third of patients develop distant metastases [5].

## Clinical case

**Figure 1.**
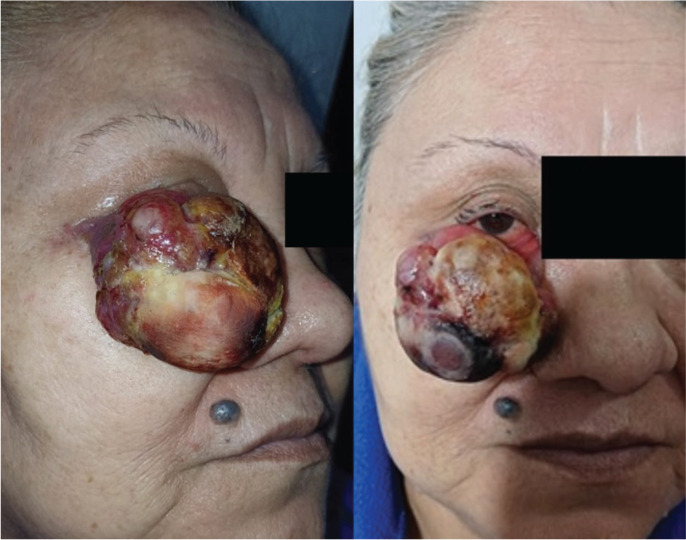
Frontal and oblique clinical photographs showing a large exophytic tumour originating from the right lower eyelid. According to the TNM classification for MCC, this corresponds to a cT2 lesion (greater than 2 cm but less than 5 cm). Based on the TNM system for eyelid carcinoma, it qualifies as cT3c (tumour >3 cm involving the full thickness of the eyelid).

A 58-year-old woman with no relevant medical history presented with a progressively enlarging tumour on the right lower eyelid first noticed 3 months prior. The lesion reached approximately 5 cm in diameter, leading to complete occlusion of the right eye and generating significant anxiety for the patient, primarily due to aesthetic concerns. Despite its size, the lesion was painless and did not compromise vision (Figure 1).

Clinically, the tumour appeared as an exophytic, firm-elastic, lobulated mass with a reddish-violet coloration and a yellowish shiny surface, superficial vascularisation, and a necrotic area in the lower portion. The borders were well-defined, with no ulceration or visible discharge.

A biopsy revealed proliferation of neoplastic cells showing diffuse and intense positivity for CK AE1/AE3, cytokeratin 20, and chromogranin, and negativity for CD20 and CD3. The Ki-67 proliferation index was 90%. These findings confirmed the diagnosis of MCC.

**Figure 2.**
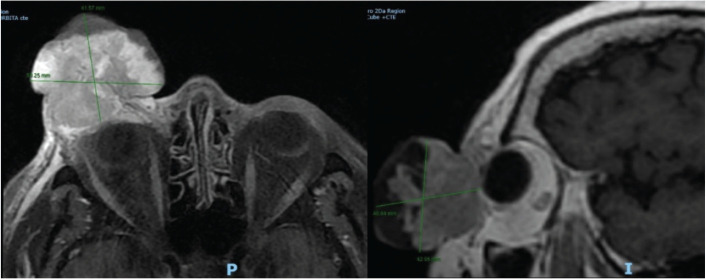
Contrast-enhanced MRI of the brain and orbits. A large exophytic mass is seen in the right orbit, located in the preseptal region and arising from the lower eyelid. The lesion shows heterogeneous contrast enhancement and is associated with ipsilateral enophthalmos due to mass effect. Its axial dimensions are approximately 45 × 38.7 mm.

Magnetic resonance imaging (MRI) with gadolinium demonstrated a large exophytic lesion in the right orbit, located in the preseptal region and arising from the lower eyelid. After intravenous contrast administration, the lesion showed heterogeneous enhancement and was associated with ipsilateral enophthalmos, consistent with mass effect. The lesion measured approximately 45 × 38.7 mm in the axial plane (Figure 2).

A contrast-enhanced computed tomography (CT) scan of the neck, thorax, abdomen, and pelvis revealed no distant metastases, although infracentimetric right cervical lymph nodes were observed.

**Figure 3.**
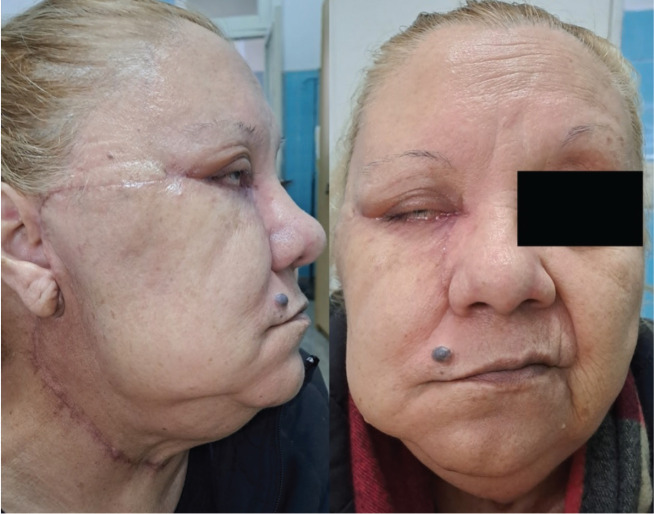
Frontal and lateral postoperative clinical photographs. The images show the surgical scar following resection of the MCC and the right cervical lymphadenectomy.

Complete resection of the tumour was performed, along with a right cervical lymph node dissection and parathyroidectomy (Figure 3). Intraoperatively, the tumour appeared well delimited, with no evidence of bony or intraorbital invasion. A wide excision with 1 cm clinical margins was achieved. Following tumour resection, a total right parotidectomy was performed with preservation of the facial nerve, confirming intraparotid metastatic involvement with macroscopically free margins. A selective neck dissection of levels I, II, and III was also carried out on the ipsilateral side. The skin and soft tissue defect of the cheek was reconstructed using a Mustardé flap, achieving adequate eyelid closure.

The histopathological analysis described a 6 × 6 × 4 cm tumour of the right lower eyelid with areas of necrosis and hemorrhage. There was evidence of vascular emboli and perineural invasion. Surgical margins were free of tumour. The right cervical dissection showed no metastases in the 27 lymph nodes analysed. However, 2 of 3 nodes in the area adjacent to the parotid gland demonstrated carcinomatous infiltration with vasculolymphatic emboli, but without capsular rupture. The final pathological staging was pT3 pN1, consistent with MCC.

Given the tumour location in the head and neck region and the large size, adjuvant intensity-modulated radiotherapy was initiated in the surgical bed and regional lymphatic drainage areas.

The patient was followed clinically and radiologically for 15 months, with no evidence of local or distant recurrence at the last follow-up. Eyelid function was preserved, with resolution of postoperative residual facial nerve paresis, and satisfactory cosmetic adaptation of the reconstructive graft.

## Discussion

Although the face is a frequent location of MCCs, eyelid tumours account for only 2.5% of cases [3]. Interestingly, women are about twice as likely as men to develop an MCC on the eyelids [3, 4].

Within eyelid tumours, the upper eyelid is the most commonly affected, as opposed to basal cell and squamous cell carcinomas [3].

The extent of disease at diagnosis, lymph node involvement (the main predictor of survival), lymphovascular invasion and infiltrative growth pattern are poor prognostic factors, as is the expression of p63 [2].

Tumour size at presentation is also a crucial factor in MCC of the periocular region and eyelids. The eighth edition (2017) of the *American Joint Committee on Cancer* (AJCC) Tumor, Node, Metastasis (TNM) system uses a tumour size of 2 cm or less to classify all lesions as T1, although no specific guidelines are provided for eyelid lesions. Specific staging of eyelid carcinoma correlates better with disease-free survival in these cases, subdividing lesions into T1 for tumours smaller than 5 mm, T2a for those between 5 and 10 mm, and T2b for tumours 10 to 20 mm (Table 1) [1, 4–6].

As for treatment, in patients with localised disease, surgical resection with negative margins of 1–2 cm radiated to the tumour is the treatment of choice [1, 2]. In tumours of the periocular region, wide local excision with 5 mm margins is recommended [3, 4, 7].

For patients at high risk of local recurrence after resection of the primary tumour, adjuvant radiotherapy is suggested. High-risk features include primary tumours of ≥1 cm, positive or close surgical margins, lymphovascular invasion, head and neck location, and immunocompromised status [1, 2].

Although there are no randomised trials supporting the role of adjuvant radiotherapy, observational studies suggest that it decreases the rate of locoregional recurrence and improves overall survival [1]. It is recommended to initiate radiotherapy within 8 weeks after surgery [1, 2].

There is no evidence for the use of chemotherapy or immunotherapy with checkpoint inhibitors in the adjuvant setting. Immune checkpoint inhibitors (ICIs) such as avelumab are indicated in metastatic or unresectable disease [1, 2].

Although neoadjuvant is not a standard of care, a phase I/II study of 39 patients with stage IIA-IVA MCC demonstrated pathologic complete responses close to 50% with two doses of Nivolumab 240 mg. This option may be an alternative mainly in patients whose surgery is borderline, always in selected cases and evaluated in a committee of experts [2, 8].

## Conclusion

MCC of the eyelid is a rare but aggressive entity, with prognosis influenced by anatomic location and tumour size. In this case, surgical excision followed by adjuvant radiotherapy was the treatment of choice due to the head and neck location, the significant size of the lesion, and the risk of locoregional recurrence. Timely and multidisciplinary management is key to improve the prognosis in this type of patient.

## Conflicts of interest

The authors declare that there is no conflicts of interest in the preparation of this manuscript.

## Funding

No funding was received for this study.

## Author contributions

All authors have contributed to the preparation of the manuscript.

## Figures and Tables

**Table 1.** Comparison between T category of MCC TNM and eyelid carcinoma TNM^1,6^

TNM for MMC, AJCC 8^va^ edition			TNM for Eyelid carcinoma, AJCC 8^va^ edition
T Category	T criterion	T category	T criterion
Tx	Primary tumor cannot be assessed	Tx	Primary tumor cannot be assessed
T0	No evidence of primary tumor	T0	No evidence of primary tumor
Tis	Carcinoma in situ	Tis	Carcinoma in situ
T1	Tumor diameter ≤2 cm	T1 T1a T1b T1c	Tumor with a greatest dimension ≤10 mm The tumor does not invade the tarsal plate or eyelid margin. The tumor invades the tarsal plate or eyelid margin. The tumor involves the full thickness of the eyelid
T2	Máximum tumor diameter >2 but ≤5 cm	T2 T2a T2b T3c	Tumor > 10 mm but ≤ 20 mm in greatest dimension Tumor does not invade the tarsal plate or eyelid margin Tumor invades the tarsal plate or eyelid margin Tumor involves full thickness of the eyelid
T3	Maximum tumor diameter >5 cm	T3 T3a T3b T3c	Tumor > 20 mm but ≤ 30 mm in greatest dimension Tumor does not invade the tarsal plate or eyelid margin Tumor invades the tarsal plate or eyelid margin Tumor involves full thickness of the eyelid
T4	The primary tumor invades fascia, muscle, cartilage, or bone	T4 T4a T4b	Any eyelid tumor that invades adjacent ocular, orbital, or facial structure Tumor invades ocular or intraorbital structures Tumor invades (or erodes through) the bony walls of the orbit or extends to the paranasal sinuses or invades the lacrimal sac / nasolacrimal duct or brain
